# Supplementary material for: Clinical Phenotype of PDE6B-Associated Retinitis Pigmentosa
Source: Int J Mol Sci. 2021 Feb 27;22(5):2374. doi: 10.3390/ijms22052374 (PMC7956818; doi:10.3390/ijms22052374)
Supplement: Supplementary file 1 [file ijms-22-02374-s001.pdf]

```
                                p.R100H                                   p.G137R
                                   |                                         |
PDE6B H.sapiens      DRCSLFMYRQRNGVAELATRLFSVQPDSVLEDCLVPPDSEIVFPLDIGVVGHVAQTKK
PDE6B M.mulatta      DRCSLFMYRQRNGVAELATRLFSVQPDSVLEDCLVPPDSEIVFPLDIGVVGHVAQTKK
PDE6B C.lupus        DRCSLFMYRQRNGVAELATRLFSVQPGSALEDCLVPPDSEIVFPLDIGVVGHVAQTKK
PDE6B B.taurus       DRCSLFMYRQRNGVAELATRLFSVQPDSVLEDCLVPPDSEIVFPLDIGVVGHVAQTKK
PDE6B M.musculus     DRCSLFMYRQRNGIAELATRLFSVQPDSLLEDCLVPPDSEIVFPLDIGIVGHVAQTKK
PDE6B R.norvegicus   DRCSLFMYRQRNGVAELATRLFSVQPDSLLEDCLVPPDSEIVFPLDIGIVGHVAQTKK
PDE6B G.gallus       DRCSLFMYRQRNGTPELATRLFNIQEGSTLEECLVSPDCEIVYPLDLGIVGYVAQTKK
PDE6B D.rerio        DRCSLFMYRQRNGIAELATRLFNVNKDSELDDCVVAPDSEIVFPLDIGIVGHVAQTKK
PDE6B X.tropicalis   DRCSLFMYRQRNGTPELATRLFNVKSNSKLEECLVPPDSEIVYPLDIGIVGHVAHTKK
PDE6A H.sapiens      DRMSLFMYRTRNGIAELATRLFNVHKDAVLEDCLVMPDQEIVFPLDMGIVGHVAHSKK
PDE6C H.sapiens      DRCSMFLCRSRNGIPEVASRLLDVTPTSKFEDNLVGPDKEVVFPLDIGIVGWAAHTKK
```

```
                                p.Y556C
                                   |
PDE6B H.sapiens      SISKGYRRITYHNWRHGFNVA
PDE6B M.mulatta      SISKGYRRITYHNWRHGFNVA
PDE6B C.lupus        SVSKGYRRITYHNWRHGFNVA
PDE6B B.taurus       SVSKGYRRITYHNWRHGFNVA
PDE6B M.musculus     SVSKAYRRITYHNWRHGFNVA
PDE6B R.norvegicus   SVSKAYRRITYHNWRHGFNVA
PDE6B G.gallus       SVSKGYRKITYHNWRHGFNVA
PDE6B D.rerio        SVSKGYRRITYHNWRHGFNVG
PDE6B X.tropicalis   AMKKGYRRVTYHNWRHGFNVA
PDE6A H.sapiens      SLSKGYRKITYHNWRHGFNVG
PDE6C H.sapiens      TVRKGYRAVTYHNWRHGFNVG
```

```
                                p.Y582H            p.D600N
                                   |                  |
PDE6B H.sapiens      LLMTGKLKSYYTDLEAFAMVTAGLCHDIDHRGTNNLYQM
PDE6B M.mulatta      LLMTGKLKSYYTDLEAFAMVTAGLCHDIDHRGTNNLYQM
PDE6B C.lupus        LLTTGKLKSYYTDLEAFAMVTAGLCHDIDHRGTNNLYQM
PDE6B B.taurus       LLMTGKLKSYYTDLEAFAMVTAGLCHDIDHRGTNNLYQM
PDE6B M.musculus     LLMTGKLKSYYTDLEAFAMVTAGLCHDIDHRGTNNLYQM
PDE6B R.norvegicus   LLMTGKLKSYYTDLEAFAMVTAGLCHDIDHRGTNNLYQM
PDE6B G.gallus       LLMTGKLKRYYTDLEAFAMVTAALCHDIDHRGTNNLYQM
PDE6B D.rerio        LLTTGKLKQYYTDLEVMAMITAGFLHDIDHRGTNNLYQV
PDE6B X.tropicalis   LLMTGKLKRYYTDLEAMAMVTAGFCHDIDHRGTNNLYQM
PDE6A_H.sapiens      LLVTGKLKRYFTDLEALAMVTAAFCHDIDHRGTNNLYQM
PDE6C_H.sapiens      LLMTGRLKKYYTDLEAFAMLAAAFCHDIDHRGTNNLYQM
```

```
                                p.D668G
                                   |
PDE6B H.sapiens      HLMDIAIIATDLALYFKKRAM
PDE6B M.mulatta      HLMDIAIIATDLALYFKKRAM
PDE6B C.lupus        HLMDIAIIATDLALYFKKRTM
PDE6B B.taurus       HLMDIAIIATDLALYFKKRTM
PDE6B M.musculus     HLMDIAIIATDLALYFKKRTM
PDE6B R.norvegicus   HLMDIAIIATDLALYFKKRTM
PDE6B G.gallus       HLMDIAIIATDLALYFKKRTM
PDE6B D.rerio        HLIDIAIIATDLALYFKKRTM
PDE6B X.tropicalis   HLMDIAIIATDLALYFKKRTM
PDE6A_H.sapiens      HMMDIAIIATDLALYFKKRTM
PDE6C_H.sapiens      HLFEVAIIATDLALYFKKRTM
```

```
                                p.D776N
                                   |
PDE6B H.sapiens      ELPKLQVGFIDFVCTFVYKEF
PDE6B M.mulatta      ELPKLQVGFIDFVCTFVYKEF
PDE6B C.lupus        ELPKLQVGFIDFVCTFVYKEF
PDE6B B.taurus       ELPKLQVGFIDFVCTFVYKEF
PDE6B M.musculus     ELPKLQVGFIDFVCTFVYKEF
PDE6B R.norvegicus   ELPKLQVGFIDFVCTFVYKEF
PDE6B G.gallus       ELPKLQVGFIDFVCTFVYKEF
PDE6B D.rerio        ELPKLQCGFIDFVCTFVYKEF
PDE6B X.tropicalis   ELPKLQCGFIDFVCTFVYKEF
PDE6A_H.sapiens      ELPKLQVGFIDFVCTFVYKEF
PDE6C_H.sapiens      ELPKLQVGFIDFVCTFVYKEF
```

Supplemental Figure S1: Comparative amino acid sequence alignment of rod photoreceptor specific PDE6B in various vertebrate species: *H. sapiens* PDE6B (NP_000274.2), *M. mulatta* PDE6B (XP_001094732.2), C. *lupus* PDE6B (NP_001002934.1), *B. taurus* PDE6B (NP_776843.1), *M. musculus* Pde6b (NP_032832.2), *R .norvegicus* Pde6b (NP_001099494.1), *G. gallus* PDE6B (XP_424876.3), *D. rerio* pde6b (XP_685002.1), *X. tropicalis* LOC100487945 (XP_002935501.2). The alignment was performed using HOMOLOGENE Multiple Alignment for PDE6B (https://www.ncbi.nlm.nih.gov/homologene). In addition, amino acid sequence of rod photoreceptor *H. sapiens* PDE6A (NP_000431.2) and cone photoreceptor *H. sapiens* PDE6C (NP_006195.3) was added for comparison. The amino acid affected by the missense mutation is highlighted in yellow and the missense mutation is given above the amino acid residue. The invariant Zn^{2+} metal binding motifs are marked in green.
